# Non-local impact of distal airway constrictions on patterns of inhaled particle deposition

**DOI:** 10.1098/rsos.241108

**Published:** 2024-11-06

**Authors:** James D. Shemilt, Alex Horsley, Jim M. Wild, Oliver E. Jensen, Alice B. Thompson, Carl A. Whitfield

**Affiliations:** ^1^ Department of Mathematics, University of Manchester, Manchester, UK; ^2^ Division of Immunology, Immunity to Infection and Respiratory Medicine, University of Manchester, Manchester, UK; ^3^ POLARIS, Imaging Sciences, Department of Infection, Immunity and Cardiovascular Disease, University of Sheffield, Sheffield, UK

**Keywords:** particle deposition, network modelling, inhaled therapies, cystic fibrosis, airway disease

## Abstract

Airway constriction and blockage in obstructive lung diseases cause ventilation heterogeneity and create barriers to effective drug deposition. Established computational particle-deposition models have not accounted for these impacts of disease. We present a new particle-deposition model that calculates ventilation based on the resistance of each airway, such that ventilation responds to airway constriction. The model incorporates distal airway constrictions representative of cystic fibrosis, allowing us to investigate the resulting impact on patterns of deposition. Unlike previous models, our model predicts how constrictions affect deposition in airways throughout the lungs, not just in the constricted airways. Deposition is reduced in airways directly distal and proximal to constrictions. When constrictions are clustered together, central-airways deposition can increase significantly in regions away from constrictions, but distal-airways deposition in those regions remains largely unchanged. We use our model to calculate lung clearance index (LCI), a clinical measure of ventilation heterogeneity, after applying constrictions of varying severities in one lobe. We find an increase in LCI coinciding with significantly reduced deposition in the affected lobe. Our results show how the model provides a framework for development of computational tools that capture the impacts of airway disease, which could significantly affect predictions of regional dosing.

## 1. Introduction

Inhaled therapies, including mucolytics and antibiotics, are commonly used to treat cystic fibrosis (CF) [1,2]. Recent evidence has also shown that inhaled gene therapies can halt or slow decline of lung function [3]. Airway constriction or blockage caused by thickened mucus layers or mucus plugging can create barriers to the effective application of inhaled therapies in CF. Typically, it is desired for inhaled drugs to be deposited evenly throughout the lungs’ conducting airways, including in the distal conducting airways. These small airways can be susceptible to constriction or blockage even in early disease [4], so delivering a sufficient dose to them, particularly in more diseased and poorly ventilated regions of the lungs, can be challenging. Barriers to effective application of inhaled therapies are not unique to CF; airway blockage can also alter patterns of inhaled particle deposition in chronic obstructive pulmonary disease (COPD) or severe asthma [5,6]. Experimental studies have shown that, while deposition is evidently reduced in blocked airways owing to a lack of ventilation through them, total lung deposition can be increased in patients with obstructive lung disease [7]. Innovation in the design of inhalers and nebulizers, and how they are administered, can make drug delivery to the lungs more efficient [8,9], but ventilation heterogeneity in patients with obstructive lung disease is still a significant barrier to uniform drug deposition throughout the lungs, particularly in the diseased airways [10,11]. Experimental techniques, such as gamma scintigraphy, can provide information on regional particle deposition in patients, including measurements such as central-to-peripheral deposition ratio [12,13]. Resolution is limited in imaging, and while new technologies are providing more detailed data on regional lung deposition, resolving particle deposition on the scale of individual small airways is beyond the capabilities of current experimental measurements. Computational particle-deposition modelling has the potential to provide detailed spatial information on patterns of local particle deposition in the small airways but, to do so, the heterogeneous ventilation induced by airway constriction must be accounted for.

Early whole-lung particle-deposition models, such as the trumpet models (e.g. [14]) and single-path models (e.g. [15]), modelled the airway tree as a one-dimensional structure, with physical properties that vary by depth in the lungs but without any other spatial heterogeneity. Attempts have been made to use similar models to investigate the effects of bronchoconstriction, where a uniform reduction in radius was applied to all airways at certain depths, leading to increased deposition in those airways [16]. More realistic, spatially heterogeneous patterns of airway constriction cannot be simulated with these models.

Multiple-path particle dosimetry (MPPD) models [17–20] are well-established computational tools that calculate regional particle deposition in an asymmetric airway tree. They have been shown to predict total deposition well in healthy adult lungs [20]. The MPPD model assumes that the flow rate through each airway is proportional to the volume of lung subtended by that airway. This means that constricting an airway would not generally reduce the predicted flow rate through that airway since the volume of airways and acini subtended by it would not necessarily change. A more sophisticated ventilation model has previously been incorporated into MPPD [21,22], which took into account lung compliance and resistance as well as the volume and capacity of the region distal to each airway when calculating the flow through it. The resistance of each airway was not directly related to its diameter, suggesting that constricting an airway may not have induced a significant change in the resistance or flow rate. The more complex ventilation model did allow for more heterogeneous ventilation of the lungs, but did not have a significant impact on lobar deposition rates in healthy lungs, leading the authors to recommend continued use of the simpler uniform ventilation model in subsequent versions of MPPD [21,22]. It has been acknowledged that using a uniform ventilation model is not likely to accurately predict deposition in diseased lungs [22,23].

Whole-lung models that combine three-dimensional computational fluid dynamics (CFD) simulations in the central airways with simpler one-dimensional models for the distal airways and acini have recently been developed [24–26]. The distal airways are treated either as several smaller trumpets [24] or multiple-path models [26]. These approaches provide models of deposition throughout the whole lung, including detailed patterns of deposition in the central airways. Airflow into each of the lungs’ lobes was inferred from experimental measurements. However, within the distal airways, the same simple ventilation models used in traditional trumpet or MPPD models were employed, so the effects of ventilation heterogeneity induced by distal airway constrictions could not easily be explored. Grill *et al*. [27] have recently developed a patient-specific particle-deposition model that uses a more sophisticated ventilation model. They validated their model’s outputs against experimental particle-deposition data from healthy subjects, showing good agreement and they simulated one example of deposition in diseased lungs by increasing the stiffness of a region of the lung to represent localized fibrosis. While more sophisticated particle-deposition models are being developed, the impacts of distal airway constriction representative of obstructive lung disease on deposition at the whole-organ scale have not yet been investigated.

Other studies have used CFD to model particle deposition (see the review by Longest & Holbrook [28]). This is typically limited to modelling a relatively small number of airways, and while relative ventilation of the lungs’ five lobes may be inferred from patient-specific imaging in some models [29,30], the small airways are not generally modelled, exhalation is generally not captured, and neither is any ventilation heterogeneity beyond differences in lobar ventilation. Some attempts have been made to incorporate bronchoconstriction in CFD models, but they have largely focused on simulating particle deposition in only two or three generations of airways [31,32]. Walenga & Longest [33] explored the effects of airway constriction in a geometry representative of the whole lungs, in which each lobe was modelled by a single path of airways, with the other airways branching off that single path being truncated. They applied a uniform constriction to all airways in their model; thorough exploration of heterogeneous patterns of distal airway constriction are beyond the current capabilities of CFD models.

Whole-lung models that can capture ventilation heterogeneity induced by airway constriction have been developed to simulate gas transport without particle deposition. Previous modelling studies [34,35] have investigated the effects of airway constriction on multiple-breath washout (MBW), a clinical measurement of ventilation heterogeneity. They showed that MBW indices are sensitive primarily to airway constriction severity corresponding to a reduction in radius of between 80 and 90%. It has been shown that variability in simulated MBW indices is also elevated when airways are constricted by similar amounts [36].

In this study, we present a whole-lung particle-deposition model, in which ventilation of the lungs is derived based on the resistance of each conducting airway and is driven by the expansion and contraction of individual acini. Transport of an inhaled gas and deposition of particles from that gas throughout the lungs are then calculated. Unlike models such as MPPD [18] that assume uniform ventilation, our model is capable of making physics-based predictions of how airway constrictions throughout the lungs lead to non-uniform, heterogeneous ventilation and the impacts this has on patterns of particle deposition. This advancement in modelling inhaled drug delivery in diseased lungs lays the groundwork for future development of patient-specific particle deposition models that can capture the effects of airway disease. In this study, we demonstrate how the predictions of particle deposition by the model can be fundamentally altered by airway constriction; the predictions provide an understanding and quantification of the physical mechanisms by which airway constriction and blockage impact patterns of deposition throughout the lungs. By simulating particle deposition in lungs in which patterns of small airway constriction representative of airway diseases such as CF have been applied, we establish that our model can make predictions that have biological plausibility, which matches with the existing understanding of how airway blockage reduces deposition to those blocked airways, and which provide detailed insight into how deposition in each individual airway in the lungs may be affected by localized airway constrictions.

An overview of the model set-up is provided in §2, with full details in the electronic supplementary material. The model can predict significant changes to patterns of ventilation and particle deposition throughout the lungs when there is bronchoconstriction. We investigate these effects by applying spatially heterogeneous patterns of distal airway constrictions, representative of airway disease. We apply several qualitatively different patterns of constriction, including constricting airways in one or two lobes only, constricting a number of localized clusters of distal airways, or constricting a number of airways distributed randomly throughout the lungs. We simulate associated clinical measures of ventilation heterogeneity (lung clearance index, LCI) and regional dosing (gamma scintigraphy).

## 2. Methods

### 2.1. Lung networks

The lungs contain a large, asymmetric network of conducting airways, with acini attached to the terminal bronchioles. We model every airway individually, and every acinus. The conducting airways are treated as rigid tubes, while the acini expand and contract as the lungs are ventilated. We use lung network geometries generated from computed tomography (CT) images of n=4 adolescents with CF but normal-range forced expiratory volume in 1 s. The CT images were collected clinically aligned with a previous lung imaging study [37]; local research governance was in place to use anonymized CT scans retrospectively for imaging research purposes. The dimensions and positions of the largest central airways are taken directly from CT, and the remaining conducting airways are generated using a volume-filling algorithm [38]. Each of the four geometries has approximately 60 000 conducting airways.

**Figure 1.**
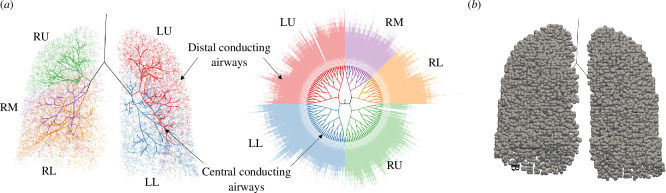
(*a*) Conducting airways in one lung network geometry, with the five lobes (right upper (RU), right middle (RM), right lower (RL), left upper (LU), left lower (LL)) highlighted. The three-dimensional network (left) is represented schematically as a planar graph (right). Path lengths from trachea (centre) to periphery are proportional to number of generations. (*b*) Acini, illustrated as spheres connected to the terminal conducting airways, for the same geometry.

Figure 1*a* shows the conducting airways in one of the geometries. We denote the generation of an airway as one more than the number of bifurcations between it and the trachea, so that the trachea is denoted generation 1. Central conducting airways, are those with generation less than 10, and distal conducting airways are the remaining conducting airways (figure 1*a*). The lungs are composed of five lobes, which are indicated in figure 1*a*. We also represent the geometry as a planar graph in figure 1*a*; we will present data from simulations below in this way to enable the visualization of deposition patterns across the entire network. We generate the planar graph representation of the network as follows. In each generation, the distal ends of the airways are placed on a circle with radius $(g-1)R_{0}$, where g is the generation and $R_{0}$ is a constant spacing. Given the angular position, $\theta_{p}$, of the distal end of an airway in generation g, the angular positions of its daughter airways’ distal ends are $\theta_{p}\pm 2^{-g}\pi$, which ensures that all airways in the same generation have the same length and that daughter airways remain adjacent to each other and connected to their parent.

To enable direct comparison of our model with MPPD, and to minimize differences purely owing to lung size, we rescale the four lung geometries so that they are representative of an adult’s lungs with functional residual capacity (FRC) of 3.3 l. Given the measured FRC, $V_{\mathrm{FRC}}$, from each geometry, we uniformly increase the length and radius of each airway in that geometry by a factor of $(3.3\;l/V_{FRC}{)}^{1/3}$. This results in a range of airway dead-space volumes between 101 ml and 145 ml, which is realistic for adult lungs, noting we do not include the oral cavity [39]. The original FRC values were between 1.1 l and 1.9 l. We focus attention on simulating deposition in the scaled lung geometries so that we can compare directly to MPPD. We do not present results from simulations in the smaller, unscaled geometries, but we found that the main qualitative difference was higher inertial impaction in the central airways in the unscaled geometries, compared to the scaled geometries, since the airway radii are smaller. Increased deposition in the central airways of children compared to adults has been highlighted in previous modelling studies (e.g. [25]). Investigating patterns of deposition in children or adolescents with CF is beyond the scope of this study. In §3.1, we present data from simulations in all four scaled geometries and compare these to MPPD to validate the model. Subsequently, we present results from simulations in one of the four geometries, chosen arbitrarily, to focus on comparing deposition in the unconstricted geometry with deposition after applying constrictions to the distal airways.

### 2.2. Ventilation

We assume that particle transport and deposition do not affect ventilation, so we first simulate ventilation and then use the calculated flow rates in each airway to simulate particle transport and deposition. The ventilation model is based on a previous model from Whitfield *et al*. [40]. We provide an overview here and details in the electronic supplementary material, S2. We represent each lung geometry as a network of edges and vertices embedded in three-dimensional space, with each edge representing an airway and vertices being placed at the ends of each edge. The resistance to airflow of each edge is calculated via Poiseuille’s law, providing a linear relationship between the difference in pressure along each airway and the flow rate through it. While Poiseuille’s law is not likely to accurately capture the complex dynamics in the largest central airways, it provides a computationally efficient simple approximation. The underlying assumption in using Poiseuille’s law is that flow in the airways is laminar, which is indeed the case in the smaller airways. However, in the largest central airways, there is likely to be turbulent flow, which is not captured by Poiseuille’s law. We have also tested the nonlinear resistance model due to Pedley *et al*. [41], which accounts for the formation of flow boundary layers at bifurcations, in simulations in healthy lung geometries but found it had minimal impact on total deposition.

When simulating ventilation, each terminal vertex of the network represents an acinus. Figure 1*b* illustrates these acini as individual spheres. We model each acinus as a viscoelastic bag, defining a relationship between the pressure in the acinus, its volume and the pleural pressure. We assume that the breathing rate at the top of the trachea and the pleural pressure both vary sinusoidally in time, and that the pleural pressure is spatially uniform. We assume a breath time of $T_{b}=5\;s$ and a tidal volume of $V_{T}=625\;ml$. All acini are assumed to have the same initial volume and compliance. The total lung elastance, combining the contributions from all acini, is taken to be $6.82{\;cm\;H_{2}O\;l}^{-1}$, which is derived from the measured value of $5{\;cm\;H_{2}O\;l}^{-1}$ for lungs with FRC of 4.5 l [42], rescaled proportionally to the FRC of 3.3 l. We tested the model after incorporating the effects of gravity on acinar dynamics by imposing a pleural pressure gradient and nonlinear compliance (adapted from [43]); while this had some effect on the fraction of particles deposited in different lobes, the effects were relatively minor and the impact on total deposition was minimal. Incorporating these gravitational effects significantly increased computational time, so they were not used.

### 2.3. Particle transport and deposition

We use the calculated air flow rates in each airway to solve an advection-diffusion equation for the transport of inhaled particles through the lungs. We solve for the concentration of inhaled particles throughout the lung network. Transport of particle concentration along an airway is governed by


(2.1) $$\begin{array}{ccc} & \frac{\partial \bar{c}}{\partial t}=\frac{\partial }{\partial z}\left(-\bar{u}\bar{c}+D_{\mathrm{eff}}\frac{\partial \bar{c}}{\partial z}\right)-s, & \end{array}$$

where z is the axial coordinate, $\bar{c}(z,t)$ is the cross-sectionally averaged concentration, $\bar{u}(t)$ is the mean velocity, $D_{\mathrm{eff}}$ is an effective diffusivity that takes into account axial diffusion and dispersion and s represents loss owing to particle deposition. To solve equation (2.1), we first discretize each airway into several edges, thus defining a modified network. Then, we recast equation (2.1) using the machinery of discrete calculus, defining a discrete analogue of the advection-diffusion equation that can be efficiently solved on the network; this combines a finite difference approximation of equation (2.1) within each airway with mass conservation for inhaled gas and particles at bifurcations between airways. Full details are given in the electronic supplementary material, S3.

**Figure 2.**
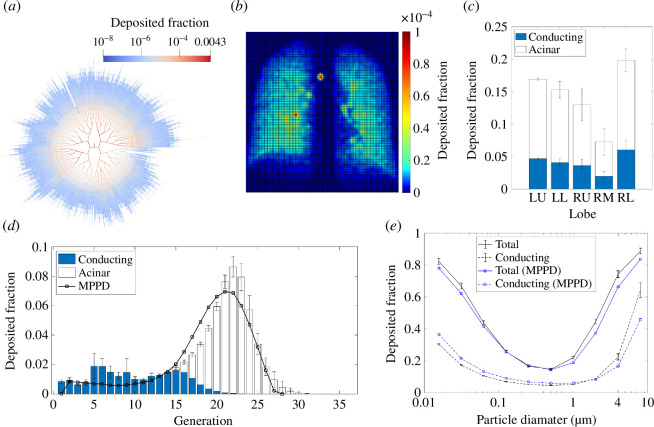
Data from simulations in the unconstricted lung geometries. In (*a*)–(*d*), particle diameter is 4 μm. (*a*) Deposited fraction within each conducting airway in one geometry, shown as a planar graph. Deposition at bifurcations is assigned to the parent airway. (*b*) Simulated scintigraphy plot for the same example. (*c*) Deposited fraction in each lobe, showing mean ± 1 standard deviation (s.d.) across the four geometries. Deposited fraction separated into conducting-airways and acinar deposition. (*d*) Deposited fraction in each generation, showing mean ± 1 s.d. across the four geometries, with conducting-airways and acinar deposition indicated. Generational deposition from MPPD is also shown. (*e*) Total and conducting-airways deposition, versus particle diameter, with comparison to MPPD.

We define s in equation (2.1) by assuming that deposition occurs via three mechanisms: inertial impaction, gravitational sedimentation and diffusion. We mostly present simulations of 4 μm diameter particles, as this is representative of the typical mass median aerodynamic diameter (MMAD) of particles generated by nebulizers used to administer inhaled therapeutics [44]. For 4 μm diameter particles, the dominant deposition mechanisms are likely to be impaction and sedimentation [23]. We also include deposition by diffusion in the model, which enables the model to describe deposition of smaller particles. In figure 2, we validate predictions against MPPD for a range of particle diameters from approximately $10^{-2}$ μm to 8 μm. Following the approach of many other studies [23], we assume that the three deposition mechanisms act independently, and we approximate deposition rates using semi-empirical or derived formulae. For impaction, we use a formula from Zhang *et al*. [45] derived from CFD simulations in airway bifurcation geometries. For sedimentation, we use a formula from Pich [46], which was also used by Oakes *et al*. [24]. For diffusion, we use a formula from Ingham [47], which has been validated against experimental results and CFD for sub-micron nanoparticles [48]. We do not model extrathoracic deposition, which is typically significant primarily for particles at least 6 μm in diameter [49], focusing instead on patterns of lung deposition.

When simulating particle deposition, each acinus is modelled as a symmetric tree of airways. Each acinar airway consists of a duct of fixed radius, and a region of alveolar space with time-varying volume. The lengths and radii of the ducts are based on data from [50], as is the relative volume of each airway within an acinar tree. The total volume of each acinus at each moment in time is taken from the ventilation simulation.

We model particle deposition during a single breath, assuming that the initial concentration of particles is zero everywhere, and that the flux of concentration into the trachea is fixed during inhalation. To solve the discrete advection-diffusion equation, we approximate the time derivative using a first-order backwards Euler finite-difference scheme, and solve the resulting system of equations using the BiCGSTAB solver in the Eigen C++ library [51]. Using an implicit, backwards finite difference scheme provides improved stability of numerical solutions compared to many explicit schemes. We use a time step of Δt=0.01s, which is small enough that total deposition is independent of the exact value. To discretize the airways to solve equation (2.1), we ensure all edges within each airway have the same length; every airway, including in the acini, contains at least eight edges and edges are at most 200 μm long. The four discretized lung networks have between 2.5 and 3 million edges each. This discretization ensures convergence of both conducting and acinar airway deposition; in the simulation presented in figure 2*a*, approximately doubling the total number of edges changes the total deposition by only 0.2%. One typical simulation takes around 2 h on a single-core 3.0GHz processor.

In figure 2, we validate the model’s output against MPPD simulations [52]. In the MPPD simulations, we use their stochastic lung geometry with an airway dead space of 113 ml, which is within the range of dead-space values of our four lung geometries. Breath time, tidal volume and FRC are all the same as outlined in §2.2, although MPPD uses a constant rate of inhalation and exhalation while we use a sinusoidal breathing profile.

### 2.4. Application of airway constrictions

We investigate the effects of airway disease by applying several different patterns of constrictions to the distal conducting airways in one of the lung geometries. We always constrict a subset of the generation 12–15 airways only, and constrict these airways all with the same severity (a value between 0 and 1, the proportion by which we reduce the airways’ radii). The way in which we select the airways for constriction, plus the constriction severity, determines the pattern of constriction. These choices are made both to illustrate key aspects of the physics of the system, and to represent typical features of CF disease. CF affects the small airways first [4], motivating our focus on distal airway constrictions. Ventilation magnetic resonance imaging (MRI) of patients with CF typically shows multiple small patches of poorly ventilated lung [37,53], motivating us to apply several localized clusters of airway constrictions in §3.3. We also investigate scenarios where constrictions are distributed randomly throughout the lungs or localized to one lobe. We use the same breath time and tidal volume before and after applying constrictions. There is evidence that, in CF, the volume of air entering the lungs over a fixed time of several breaths does not change significantly as disease severity increases [54], although breathing may become faster and shallower in very severe disease. Mild disease, which we focus on simulating here, is not likely to significantly affect breathing rates, and keeping breathing parameters fixed allows us to focus on the impacts of airway constriction.

### 2.5. Simulated multiple-breath washout and gamma scintigraphy

We modify the model to also simulate MBW. We simulate many breaths instead of one, set the deposition term in equation (2.1) to zero, set the diffusivity to the value for nitrogen gas in oxygen at 37°C, and assume an initially uniform concentration of tracer gas (nitrogen) throughout the lungs. We also assume that gas is well-mixed within each acinus, as the diffusivity of nitrogen is much higher than that of any sized aerosol we consider in the deposition model, so acinar mixing is likely to be much stronger. This MBW model is effectively equivalent to that of Foy *et al*. [34]. LCI, a clinical measure of ventilation heterogeneity, is then calculated as follows. Suppose that after $n_{L}$ breaths, the concentration at the top of the trachea, $c_{0}(t)$, at end-exhalation is less than 1/40 of the initial concentration, $c_{0}(n_{L}T_{b})<c_{0}(0)/40$ and that this is not the case for the previous breath. Then


(2.2) $$\begin{array}{ccc} & \mathrm{LCI}=\frac{V_{\mathrm{ce}}}{V_{\mathrm{tr}}}\left[c_{0}(0)-c_{0}(n_{L}T_{b})\right], & \end{array}$$

where $V_{\mathrm{ce}}$ is the cumulative expired volume of gas and $V_{\mathrm{tr}}$ is the total volume of exhaled tracer gas [55].

We use particle-deposition predictions to simulate gamma scintigraphy, a clinical measurement of regional dosing, which uses radiolabelling to image the distribution of particles in real patients. To do so, we divide the two-dimensional plane into many pixels, sum the deposition that occurs within each pixel (across the whole depth of the lungs) and use kernel density estimation in MATLAB to generate a deposition distribution that mimics a scintigraphy image. The simulated scintigraphy is therefore derived entirely from the particle-deposition calculations, and is simply a method for visualizing the simulation outcomes. Further details of the process of generating a simulated scintigraphy image are in the electronic supplementary material, S5.

## 3. Results

### 3.1. Particle deposition in the lungs without airway constriction

We first simulate particle deposition in all four lung geometries without applied bronchoconstriction, and compare results to MPPD for validation (figure 2). Deposition is typically much higher in larger central airways than in smaller distal airways, but there is some heterogeneity in the spatial distribution of deposition owing to asymmetries in the lung geometry (figure 2*a*). There is a range of path lengths between the trachea and the terminal conducting airways: in the geometry in figure 2*a*, the shortest path terminates at generation 8, and the longest paths extend beyond generation 20. Deposition is generally higher in regions where path lengths are longer (figure 2*a*). Since each terminal conducting airway connects to exactly one acinus, regions with a lot of long paths have a larger number of acini, so more particles are typically drawn through them and so more deposit. Simulated scintigraphy (figure 2*b*) demonstrates how the model can reproduce features of healthy deposition patterns: for example, deposition is relatively uniform spatially, with a few hot spots at central-airway bifurcation points where significant inertial impaction occurs. It shows higher deposition in the centre of the image where the lung is thicker, so the cumulative deposition across the whole depth of the lungs is higher. Figure 2*c* shows the distribution of deposited particles between the lungs’ five lobes.

To validate the model in healthy lung geometries, we compare deposition in the four lung geometries against results from MPPD (figure 2*d,e*). Deposition in each airway generation shows generally good agreement for 4 μm diameter particles (figure 2*d*), despite MPPD using a lung geometry based on different morphometric data. There is also good agreement with MPPD predictions of total deposition over a range of particle sizes, with only minor quantitative differences for particle diameters smaller than 0.1 μm or larger than 1 μm. Higher conducting-airways deposition for large particles in our model is probably owing to the central airways being narrower on average than in the MPPD lung geometry, causing higher impaction. We use different semi-empirical formulae than in MPPD to determine deposition rates, but despite this, agreement is still good.

The MPPD model has been validated against experimental data in people with healthy lungs [56], showing good agreement in total deposition over a range of particle sizes from 0.01 μm to 10 μm diameter particles [20,22]. Good agreement (figure 2*d,e*) between our predictions of lung deposition and MPPD, therefore, also indicates that our model accurately captures lung deposition fractions across a wide range of particle sizes in healthy lungs. Comparison to the MPPD model provides validation in healthy lung geometries, but the MPPD model assumes a uniform ventilation so it is not able to capture the effects of airway constriction or blockage on patterns of ventilation [22,23]. In the following sections, we demonstrate our model’s ability to predict heterogeneous patterns of ventilation and particle deposition after many airways in the lungs have been constricted.

### 3.2. Effects of varying airway constriction severity

**Figure 3.**
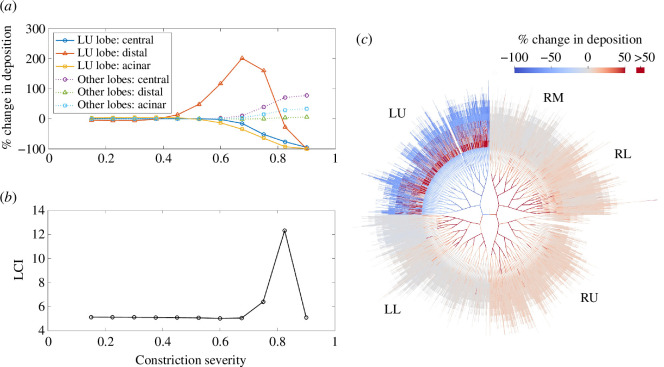
Simulations in which all generation 12–15 airways in the left upper (LU) lobe are constricted with varying severities (fraction by which airway radii are reduced). In each simulation, the constriction severity is the same for all LU lobe airways. (*a*) Change in deposition in the affected lobe (LU) and the other lobes, separated into central conducting-airways deposition, distal conducting-airways deposition and acinar deposition. (*b*) Lung clearance index (LCI), from MBW simulations in the same constricted geometries. (*c*) Change in individual airway deposition in the simulation with constriction severity 0.825.

To explore how the severity of airway constriction impacts deposition, we first constrict all generation 12–15 airways in a single lobe, with a range of constriction severities. The effect of increasing constriction severity on deposition in the affected airways is non-monotonic (figure 3*a*). In these simulations, distal-airways deposition is not found to be significantly impacted by mild constrictions but, when the severity exceeds 0.4, it increases as impaction is significantly enhanced in the narrowed airways. As the severity is increased further beyond 0.675, the increased resistance of the affected airways causes flow to be reduced into the affected lobe to such a degree that distal deposition decreases rapidly. Increased impaction in narrowed airways has been highlighted previously in simpler models [16], but these have not captured the subsequent decrease as airway resistance is further increased and ventilation patterns are altered.

Central and acinar deposition in the affected lobe decreases as the constriction severity is increased and ventilation of the lobe is reduced (figure 3*a*). In the other lobes, deposition increases owing to a higher proportion of the inhaled particles entering these lobes and the speed of air flow through them being faster.

The strongest increase in deposition in the unconstricted lobes is in the central airways since impaction is the dominant deposition mechanism there, and this is enhanced by faster flow. By contrast, in the distal conducting airways, where sedimentation is the dominant mechanism, there is minimal change to deposition as shorter residence times for particles lead to reduced sedimentation, enough to largely offset any increased impaction. The changes in deposition that we predict in airways that are not themselves constricted cannot be reproduced by existing models such as MPPD [18], which assume uniform ventilation. Figure 3*a* shows that when the left upper lobe airways are severely constricted, we predict up to an 80% increase in central-airways deposition in the rest of the lungs, and almost no change in distal-airways deposition. This quantification of the relative impact on deposition in the central and distal airways suggests a mechanism for how localized airway constriction can increase the central-peripheral deposition ratio throughout the lungs, a measure that can be increased [12,13] in people with obstructive airway diseases, albeit with significant inter-subject variability in the experimental data.

As the constriction severity is increased in these simulations, other parameters such as particle size and breathing rate are kept constant, so as to isolate the impacts of airway constriction. The assumption of fixed tidal volume means that local changes in flow rate are solely owing to redistribution of lobar ventilation. Some of the changes in regional deposition might be partially mitigated by adjusting these other parameters: for example, since impaction is weaker for smaller particles and at lower flow rates, the increase in central-airways deposition after applying severe constrictions may be smaller if the particle size or breathing rate was decreased. However, in the simulations shown in figure 3, we assume a typical flow rate for normal breathing and a particle size of 4 μm, which is typical of the MMAD of many nebulized drugs.

Figure 3*b* shows how LCI responds to the same applied constrictions. In agreement with previous modelling studies [34–36] we find that LCI is sensitive to a narrow range of constriction severities, which correspond to a reduction of airway radius of around 80%. Comparison with the deposition results (figure 3*a*) indicates that the increase in LCI coincides with where deposition is strongly reduced in the central and acinar airways in the constricted lobe, and increased elsewhere. When constriction severity is 0.825, there is significantly reduced flow through the left upper lobe, but many of the constricted airways still receive increased deposition (figure 3*c*). The model predicts that when LCI is raised, particle deposition is likely to be more heterogeneous owing to altered ventilation patterns, but constricted airways can still receive a significant dose. However, airways proximal or distal to those severely constricted airways are likely to receive a reduced dose, and a large fraction of the inhaled dose may be diverted to unconstricted regions of the lung. In keeping with previous modelling results, figure 3*b* suggests that LCI is mainly sensitive to severe (but not total) airway constrictions. Deposition of 4μm-diameter particles is, however, sensitive to a wider range of constriction severity, with contrasting effects from mild and severe airway constrictions.

### 3.3. Effects of varying spatial patterns of airway constriction

**Figure 4.**
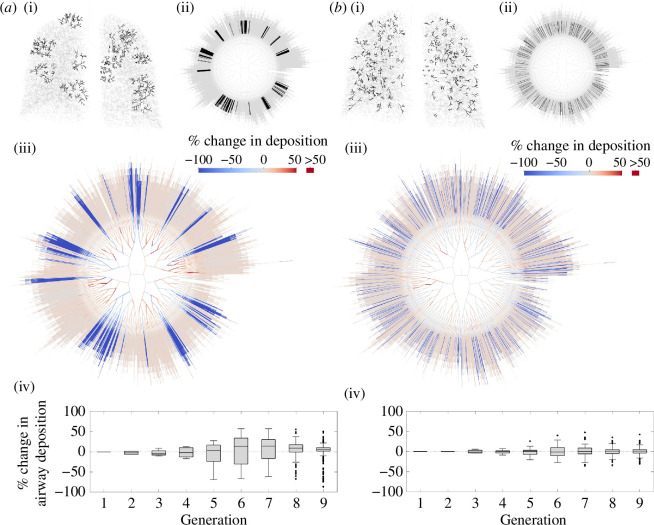
Comparison of two patterns of distal airway constrictions. In (*a*), constrictions (severity 0.9) are applied in 12 clusters. Each cluster is generated by randomly selecting a generation-12 airway, then constricting all generation-12 airways within a radius of $R_{C}=2.4\;cm$ of it and all of their descendants down to generation 15. We enforce that no clusters overlap. In total, 322 generation-12 airways, and all of their descendants down to generation 15, were constricted. In (*b*), 322 generation-12 airways were chosen at random, and they and their descendants down to generation 15 were constricted (severity 0.9). (i) Constricted airways highlighted in the three-dimensional networks, and (ii) in the planar graph representations. (iii) Change in airway deposition versus deposition in the unconstricted geometry. (iv) Change in individual airway deposition as box plots for generations 1–9.

**Figure 5.**
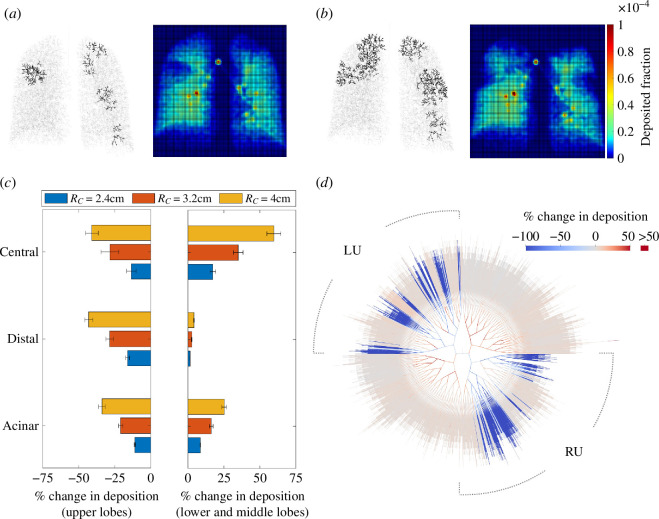
Data from simulations with six clusters of distal airway constriction (severity 0.9) in the upper lobes. In each simulation, six generation-12 airways were selected at random in the upper lobes, and all airways within a radius of $R_{C}$ of any of these were constricted, along with all of their descendants down to generation 15. Four realizations were simulated for each of $R_{C}=2.4\;cm$, $R_{C}=3.2\;cm$ and $R_{C}=4\;cm$. (*a*) An example with $R_{C}=2.4\;cm$, and (*b*) an example with $R_{C}=4\;cm$, showing constricted airways (left) and simulated scintigraphy (right). (*c*) Percentage change in deposition versus the unconstricted case, showing change in the upper lobes (left), and in the other lobes (right). These data are also separated into central conducting, distal conducting and acinar deposition. Mean ± standard deviation across the four realizations is plotted for each case. (*d*) Change in individual airway deposition for an example with $R_{C}=3.2\;cm$.

To investigate the impact of the spatial distribution of distal airway constrictions, we simulate two patterns of applied airway constrictions: clustered constrictions (figure 4*a*) and constrictions distributed randomly throughout the lungs (figure 4*b*). To generate clusters of constriction, we randomly select $N_{C}=12$ generation-12 airways, then constrict all generation-12 airways within a radius of $R_{C}=2.4\;cm$ of any of these airways, and constrict all of their descendants down to generation 15. We apply severe constrictions (severity 0.9) to all of these airways. The same process is also used to generate clustered constrictions in figure 5, but the number of clusters, $N_{C}$, and the radius, $R_{C}$, are different. We always enforce that no two clusters overlap.

Both for clustered and randomly distributed constrictions, deposition is reduced significantly in the constricted airways, and in the airways distal to the constrictions, since flow through them is strongly reduced (figure 4*a*(iii),*b*(iii)). Deposition is also reduced in the airways directly proximal to the constricted airways. This effect is stronger when multiple constricted airways are clustered together (figure 4*a*(iii)), with decreases in deposition extending more proximally and the decreases being stronger than when constrictions are not clustered (figure 4*b*(iii)). This difference is also evidenced by comparison of figure 4*a*(iv),*b*(iv): more airways in generations 5–9 have significantly reduced deposition when constrictions are clustered than when they are not.

Both when the constrictions are clustered and when they are not, deposition typically increases in airways that are not directly proximal or distal to a constriction (figure 4*a*(iii),*b*(iii)). This is because more of the inhaled gas is transported through these open paths once other paths are blocked, and flow through them is faster, so inertial impaction increases. When constrictions are clustered, increases in deposition in the central airways are generally larger than when constrictions are not clustered. This is evident in the comparison of figure 4*a*(iii),*b*(iii), and of figure 4*a*(iv),*b*(iv). Figure 4*a*(iv) shows positive, although relatively small, median increases in airway deposition in several generations of central airways. Figure 4*b*(iv) shows almost exactly zero median change in the same generations when constrictions are not clustered. Variability in deposition in almost every generation of central airways is larger when constrictions are clustered. This highlights how clustered constrictions in the distal airways can cause more heterogeneous central-airways deposition.

To examine the impact of increasing the size of clusters of constriction, we now apply $N_{C}=6$ clusters to the upper lobes, and vary the cluster radius, $R_{C}$ (figure 5). The process of generating clusters of constrictions is otherwise as described above. Since $N_{C}$ is kept fixed, increasing $R_{C}$ typically leads to a marked increase in the number of airways being constricted, so that the simulations with higher values of $R_{C}$ are representative of more severely diseased states compared to those with lower values of $R_{C}$. Simulated scintigraphy can be seen to clearly detect large clusters of constriction where many airways are blocked (figure 5*b*). Where clusters of constrictions are smaller and fewer airways are constricted, not all constricted regions are visible on simulated scintigraphy (figure 5*a*). In figure 5*a*, a patch of reduced deposition is clearly visible in the right lung, where two clusters are close together, but small clusters on the left lung, particularly those occurring in the centre of the image where lung depth is thickest, are not clear on the simulated scintigraphy. This highlights potential limitations of scintigraphy: it is more sensitive to severe blockages, and it may be more sensitive to constrictions that occur on the edges of the image field than those that occur in the centre.

Figure 5*c* quantifies how deposition changes in different regions of the lungs as the size of cluster of constrictions is increased. In the upper lobes, deposition decreases in the central, distal and acinar airways as the clusters are made larger and more airways are constricted. Elsewhere, deposition is increased as flow becomes faster through the unconstricted lobes. Central-airways deposition increases significantly, while there is almost no change to deposition in the distal conducting airways in the unconstricted lobes. Impaction is the dominant mechanism in the central airways, so deposition there is much more sensitive to increased flow rates. In fact, figure 5*c* suggests that the increase in deposition in the central airways in these lobes is at least as large as the decrease in the lobes with constrictions. Within the constricted lobes, there are still some open paths, and deposition in airways on these paths still tends to increase as long as they are not directly proximal to a constriction (figure 5*d*). Therefore, some inhaled particles continue to be deposited in a lobe even if many of its airways are constricted, although deposition then strongly favours the open airways that are not on the same path as any constriction. The lack of increase in distal-airways deposition in the lobes without constrictions (figure 5*c*) is owing to the fact that increased flow rates mean shorter residence times for most inhaled particles, so decreased sedimentation, which is the dominant deposition mechanism in the distal airways. In turn, this means residence times in the acini are longer in these lobes, leading to increased acinar deposition. In these lobes, the ratio of central to distal conducting airway deposition is increased purely by the application of constrictions elsewhere in the lungs.

## 4. Discussion

We have developed a model of inhaled particle deposition that predicts ventilation patterns based on the resistance of every airway in the lungs. Existing computational models, such as the MPPD model [18], have been built on assumptions that the lungs are healthy and that ventilation is uniform. Such models cannot predict the impacts of airway constriction and blockage on ventilation and particle deposition. Our model, therefore, provides a first step towards predicting particle deposition in patients with obstructive lung disease. The model outlined here provides a framework that can in future be extended to incorporate more clinically relevant physics, and to integrate clinical measurements of lung function and ventilation heterogeneity to move towards patient-specific predictions of drug deposition in obstructive lung diseases.

We have tested the model in several lung geometries, validating total deposition results in healthy lung simulations against MPPD [52], showing good agreement even despite using different lung geometries. We investigated the impact of applying various patterns of distal airway constrictions, representative of features of CF disease. Strong changes in deposition can occur in the constricted airways themselves, but also, when changes to ventilation are taken into account, there can be significant impacts on deposition in unconstricted airways elsewhere in the lungs. Deposition in airways directly proximal or distal to constricted airways is reduced as flow rates through these paths are decreased. The severity of constriction at which this decrease becomes severe approximately coincides with a spike in the predicted LCI. We demonstrated that when distal airway constrictions are clustered together, their effects on deposition in the central airways can be heightened, with deposition becoming more heterogeneous. Central airways away from the constrictions received significantly increased deposition, driven by enhanced inertial impaction as flow rates were increased through these open paths, since constricted airways received less flow and the flow rate into the trachea was assumed to be the same before and after applying constrictions. By contrast, the unconstricted distal conducting airways tended not to receive an increased dose since gravitational sedimentation, the dominant deposition mechanism in the small airways, is not increased by the faster flow. This disparity between the impact on central-airways and distal-airways deposition away from constrictions highlights a mechanism by which an increase in central-to-peripheral deposition ratio may be driven by the presence of localized severe distal airway constrictions, and our simulation predictions provide some quantification of this effect.

Our results highlight the importance of accounting for altered ventilation patterns when simulating particle deposition in lungs affected by airway disease. Experimental studies have previously established that CF disease can significantly alter sites of particle deposition [7]. We found that severe constrictions in the lungs’ upper lobes, which are often affected early in CF [57], can cause increased deposition in the central airways elsewhere in the lungs. This may partially explain the increased central-airways deposition observed in some severe CF patients [7]. However, other effects, such as increased turbulence, which our model cannot account for, may also contribute. Further work directly replicating the experiments of Anderson *et al*. [7] would be required to confirm this. In severe disease, breathing may become faster and shallower [54], which may also enhance central-airways deposition.

Our model provides detailed predictions of deposition patterns throughout the central and distal airways, which have a significantly finer resolution than can be achieved with current medical imaging techniques. The physical insights we have provided, relating changes in ventilation and deposition patterns, have the potential to improve estimates of regional dosing of an inhaled therapeutic once ventilation patterns have been inferred in a patient’s lungs from clinical tests of ventilation heterogeneity, such as MBW or ventilation MRI [53,55]. Dosing plans drawn up based on lung exposure calculated from MPPD and healthy volunteer studies may not be accurate for those with significant ventilation heterogeneity. Our data suggest there may be particular risks around higher concentrations of inhaled drugs being delivered to central airways and lower doses to the most strongly affected lung regions.

Some relevant physical effects are not included in the current model, but could be incorporated in future: for example, airway wall elasticity [10], which could enable modelling of transient airway collapse; mechanical interdependence of the acini, which has been incorporated in previous ventilation models [58] and extrathoracic deposition, which may be significant for large particles [5]. Incorporating extrathoracic deposition could enable direct comparison with experimental data in patients; here, we have compared deposition results against MPPD simulations without extrathoracic deposition, which still provides good validation since MPPD compares well with experimental data [20,22]. To account for the effects of boundary-layer phenomena, plug-like flow, or the increase in resistance owing to turbulent dissipation, particularly in large airways, Poiseuille’s law could be replaced by an alternative resistance law [41,59–61]. The impacts of altered airway wall mechanics on particle deposition may be particularly relevant if extending the model to describe drug delivery in asthma [10] and incorporating spatially varying elastic properties into the acinar model could enable modelling of heterogeneous emphysema in COPD [62]. Developing the model in future to describe the formation of airway blockages, or to capture the changes in airway wall mechanics associated with disease [10], could allow for investigation of more complex airway dynamics than the case of fixed airway constrictions considered here. These extensions could enable the model to describe a wider range of diseases and disease states in future.

We have provided detailed physical insight into the effects of simulated patterns of airway constriction, representative of typical features of CF. In order to isolate the impacts of airway constriction, we have not varied parameters such as breathing rate or tidal volume. Faster, shallower breathing should be incorporated if modelling patients or patient-phenotypes where this is observed [54]; this study focused on features representative of mild disease, where breathing rates are unlikely to be significantly altered. We have demonstrated that the model can simulate deposition of particles of a wide range of sizes (figure 2*e*), but have focused on assessing the impacts of airway constriction on deposition of 4μm-diameter particles. Systematic investigation of the effects of varying particle size in lung models with airway constrictions is left for a future study. By assessing the impacts of breathing rates and particle size in diseased lungs, modelling results could then be used to suggest new clinical strategies for drug delivery.

The model presented here provides an advance on previous models, such as single-path [15] or multiple-path [18] models, by incorporating a physics-based ventilation model that accounts for the resistance of each individual airway. This means that it can predict the impacts of spatially heterogeneous patterns of airway constrictions or blockages on ventilation and particle deposition throughout the lungs. Models incorporating three-dimensional CFD simulations in the central airways provide more detailed predictions of deposition patterns, but the computational cost of CFD generally means only a small number of airways can be simulated [28]. Experimental and numerical studies have shown that turbulence in the upper airways can enhance rates of particle deposition [63], and CFD models may be able to capture these effects. Models that couple CFD in the large airways with reduced-order models for the distal airways can simulate deposition throughout the lungs on inhalation and exhalation (e.g. [24,26]), but the whole-lung models of Oakes *et al*. [24,25] or Kuprat *et al*. [26] assume uniform ventilation within the distal airways, so these models could not have responded to distal airway constrictions in a physics-based way, as our model does. Our reduced-order modelling approach lowers the computational time for our simulations compared to most CFD models, enabling us to run a larger number of simulations to investigate different patterns of airway constriction.

Using a novel computational model that accounts for altered ventilation induced by airway constrictions, we have demonstrated some of the key impacts of airway disease on patterns of inhaled particle deposition. We have demonstrated how airway constrictions localized to the distal airways can affect deposition throughout the lungs. Spatial clustering of constrictions can increase the impact on central-airways deposition, making it more heterogeneous by reducing deposition in airways directly proximal to constrictions and increasing deposition elsewhere. These results have implications for understanding how ventilation defects and airway blockage in obstructive lung diseases may affect how inhaled therapeutics deposit. This is an important step towards developing realistic simulations of particle and drug deposition that can be used to accurately model exposure in disease states as well as healthy lungs.

## Data Availability

The code used to simulate ventilation and particle transport and deposition is available via [64]. Code used to generate airway constriction patterns, the network files used in all simulations and the code used to generate simulated scintigraphy plots are available via [65]. Generation of the lung networks used the same method as Whitfield *et al*. [40]; the code used to generate those networks was published previously here [66], with the airway centre-line data from CT and computed networks here [67]. Supplementary material is available online [68].
